# A structural equation model of CFIR inner and outer setting constructs, organization characteristics, and national DPP enrollment

**DOI:** 10.1186/s43058-023-00522-3

**Published:** 2023-11-17

**Authors:** Lillian Madrigal, Regine Haardörfer, Michelle C. Kegler, Sarah Piper, Linelle M. Blais, Mary Beth Weber, Cam Escoffery

**Affiliations:** Rollins School of Public Health Emory University, 1518 Clifton Rd, Atlanta, GA 30322 USA

**Keywords:** Chronic disease, Prediabetes, Implementation science, Program evaluation, Program research, Consolidated Framework for Implementation Research, Structural equation modeling

## Abstract

**Background:**

The National Diabetes Prevention Program (DPP) has made great strides in increasing accessibility to its year-long, evidence-based lifestyle change program, with around 3000 organizations having delivered the program. This large dissemination effort offers a unique opportunity to identify organization-level factors associated with program implementation and reach (enrollment) across diverse settings. The purpose of this study was to quantitatively examine the relationships among Consolidated Framework for Implementation Research (CFIR) *Inner Setting* and *Outer Setting* constructs and the implementation outcome of reach.

**Methods:**

This study analyzed data from a 2021 cross-sectional online survey with 586 National DPP Staff (lifestyle coaches, master trainers, program coordinators) with information about their organization, implementation outcomes, and responses to quantitative CFIR *Inner Setting* and *Outer Setting* construct items. Structural equation modeling was used to test a hypothesized path model with *Inner* and *Outer Setting* variables to explore direct and indirect pathways to enrollment.

**Results:**

The CFIR items had good internal consistency and indicated areas of implementation strength and weakness. Eight variables included as part of the CFIR structural characteristics and one organization characteristic variable had significant direct relationships with enrollment. The length of delivery, number of lifestyle coaches, number of full-time staff, large organization size, and organizations delivering in rural, suburban, and/or urban settings all had positive significant direct relationships with enrollment, while academic organizations and organizations with only non-White participants enrolled in their National DPP lifestyle change programs had a negative association with enrollment.

**Conclusions:**

Participant reach is an important implementation outcome for the National DPP and vital to making population-level decreases in diabetes incidence in the USA. Our findings suggest that to facilitate enrollment, program implementers should focus on organizational structural characteristics such as staffing. Strengths of this study include the use of adapted and newly developed quantitative CFIR measures and structural equation modeling. Health prevention programs can use the methods and findings from this study to further understand and inform the impact of organization factors on implementation outcomes.

**Supplementary Information:**

The online version contains supplementary material available at 10.1186/s43058-023-00522-3.

Contributions to the literature

- This work builds upon the CFIR literature and provides new understanding of implementation science measures.
- Health prevention programs can use the methods and findings from this study to further understand and inform the impact of organization factors on implementation outcomes.
- The length of delivery, number of lifestyle coaches, number of full-time staff, large organization size, and organizations delivering in rural, suburban, and/or urban settings all had positive significant direct relationships with enrollment.

## Background

Currently, 96 million (38%) US adults have prediabetes, a condition where blood glucose levels are higher than normal, but not high enough to be diagnosed with type 2 diabetes [1]. To address prediabetes, the CDC’s National Diabetes Prevention Program (DPP) has made great strides in raising awareness for and accessibility to its year-long, evidence-based lifestyle change program [2–4]. The goal of this initiative is to create public and private organization partnerships to deliver programs in communities across the country [5].

As of March 2022, there were over 600,000 participants reached by the National DPP since February 2012 [5]. Even with this success, others have estimated that in order to have population-level impact the initiative should aim to enroll 12 million people with prediabetes [6]. Additional efforts are needed to scale and sustain National DPP delivery across the country to this level. While there have been around 3,000 organizations who have delivered the program [2], very little implementation research has been done to understand organization-level factors and characteristics associated with program implementation and reach (enrollment).

The Consolidated Framework for Implementation Research (CFIR) is a meta-theory comprised of constructs that have been associated with effective implementation [7]. CFIR constructs have been useful in understanding implementation in a wide range of interventions, settings, and research designs [8–10]. In particular, the *Inner Setting* and *Outer Setting* focus on internal and external influences on organizations related to program implementation. The *Inner Setting* domain constructs aim to capture the complexity within the organization related to implementation. These include constructs such as an organization’s *structural characteristics*, *culture*, and *implementation climate*. The *Outer Setting* constructs provide insight into the greater environments and external context which constrain organizations or facilitate their ability to carry out the intervention. These include constructs such as *cosmopolitanism*, *patient needs and resources*, and *external policies and incentives* (see Table 1 for construct definitions). As part of a mixed methods study to evaluate the National DPP implementation, we applied CFIR’s *inner and outer setting* constructs to describe these internal and external organization influences on enrollment.

**Table 1:** Adapted and created CFIR items

CFIR constructs	Construct definition	Adapted/created items
*Inner Setting*		
Structural characteristics	The social architecture, age, maturity, and size of an organization	19 organizational characteristics variables
Networks and communications	The nature and quality of webs of social networks and the nature and quality of formal and informal communications within an organization	4 items adapted from the Organizational readiness for change (Helfrich, Li, Sharp, & Sales, 2009) 1 item created
Culture	Norms, values, and basic assumptions of a given organization	3 items adapted from Inner Setting Measures for Culture (Fernandez et al., 2018) 1 item created
Implementation climate	The absorptive capacity for change, shared receptivity of involved individuals to an intervention, and the extent to which use of that intervention will be rewarded, supported, and expected within their organization	4 items adapted from Inner Setting Measures for Implementation Climate (Fernandez et al., 2018) 2 items created
Readiness for implementation—leadership engagement	Commitment, involvement, and accountability of leaders and managers with the implementation	5 items adapted from Inner Setting Measures for Leadership Engagement (Fernandez et al., 2018)
Readiness for implementation—available resources	The level of resources dedicated for implementation and on-going operations, including money, training, education, physical space, and time	4 items adapted from Inner Setting Measures for Available Resources (Fernandez et al., 2018) 2 created
*Outer Setting*		
Patient needs and resources	The extent to which patient needs, as well as barriers and facilitators to meet those needs, are accurately known and prioritized by the organization	3 items adapted from Escoffery et al. 2018 2 items created
Cosmopolitanism	The degree to which an organization is networked with other external organizations	4 created
External policy and incentives	A broad construct that includes external strategies to spread interventions, including policy and regulations (governmental or other central entity), external mandates, recommendations and guidelines, pay-for-performance, collaboratives, and public or benchmark reporting	1 item adapted from Escoffery et al. 2018 2 items created

The CFIR *Inner and Outer Setting* constructs have been found to be important factors for outcomes like program delivery and scaling across different health promotion topics and settings. In 2018, a systematic integrative review identified influential organizational contextual features of healthcare settings on the implementation of evidence-based practices [11]. Organizational characteristics and *Inner Setting* constructs, *Culture* and *Leadership Engagement*, were often interrelated and worked synergistically to influence implementation outcomes (adoption, integration, and intervention use) across the 36 studies included in the review. *Inner Setting* constructs, *Leadership Engagement*, *Tension for Change*, and *Access to Information and Knowledge* were also found to be influential in an evaluation of the implementation of breast and colorectal cancer screening across a number of evidence-based practice delivery sites [12]. In a mixed-methods analysis of facilitators and barriers to scaling up tobacco control programs, CFIR *Inner Setting* constructs identified the importance of leadership engagement at multiple levels, compatibility/program fit, and adequate training/skills of staff [13].

The *Outer Setting* is also of particular importance when implementing prevention programs nationally like the National DPP, as these initiatives rely on partnerships between multiple agencies, leaders, funders, and policy makers, across national, state, and local levels [14]. In a systematic review of influential CFIR constructs on the implementation of e-health interventions, many studies included *Inner* and *Outer Setting* constructs [15]. Within the *Outer Setting*, *External Policy and Incentives* were most frequently identified as impacting implementation of e-health interventions due to effects of legislation, policies, and liability concerns on intervention delivery and the incentives by the government to facilitate intervention adoption [15]. While there are examples of how these constructs impact implementation outcomes, there is little in the literature around the relationship between the CFIR *Inner and Outer Setting* constructs and any relationships they have with each other.

In a 2022 update of the CFIR, the originators provide implementation outcome definitions categorized into anticipated (adoptability, implementability, sustainability) and actual outcomes (adoption, implementation, sustainment) [10]. In this paper, the authors also note that many applications of CFIR have been combined with other implementation frameworks that include implementation outcomes, such as the Reach, Effectiveness, Adoption, Implementation, and Maintenance (RE-AIM) [16] and the Implementation Outcomes Framework [17]. The new CFIR outcomes encompass many of the specific implementation outcomes previously identified in the literature (e.g., acceptability, adoption, fidelity, penetration, etc.). For this study, we used the RE-AIM framework and focused on the implementation outcome of reach defined as “the absolute number, proportion, and representativeness of individuals who are willing to participate in a given initiative, intervention, or program” [18], operationalized using program participant enrollment numbers.

While past CFIR research has been largely qualitative and focused on implementation at the time of adoption [8], more recent work used quantitative methods to understand the range of implementation outcomes. Quantitative measures have been used to understand the implementation and scale up of tobacco control programs, a mobile health platform, a telemedicine-delivered healthy lifestyle program, and colorectal cancer screening practices [13, 19–22]. In 2018, Fernandez and colleagues published items for five *Inner Setting* constructs (culture, implementation climate, learning climate, leadership engagement, and available resources). These items were found to have good psychometric properties and have been used by others, including to evaluate implementation barriers and facilitators of a telemedicine-delivered healthy lifestyle program [22]. For the *Outer Setting*, a recent systematic review has identified 20 measures for the various constructs; however, the majority of scales and subscales did not have psychometric information available [14].

Within the broad diabetes prevention program literature, a few studies have been published on CFIR to evaluate program implementation. In 2015, CFIR was used to systematically assess contextual factors that influence RE-AIM domains of an adaptation of the DPP for US Veterans [23]. The study identified a number of facilitators and barriers associated with CFIR *Outer* and *Inner Setting* domains; however, they are not described in depth. More recently, a number of diabetes preventions programs have been evaluated qualitatively using CFIR [24–28]. In one study, two *Outer Setting* constructs, *Peer Pressure and Cosmopolitanism*, were heavily discussed, emphasizing the benefits of the competitive edge the program gives organizations and strong partnerships [24]. Almost all constructs within *Inner Setting* were salient in the data; examples of important constructs included *Implementation Climate-Compatibility* which focused on fit within the organization and staff capacity, as well as Readiness for Implementation, which discussed the importance of *Leadership Engagement*, *Available Resources*, and staff *Access to Knowledge and Information*. Due to the qualitative nature of these studies within the diabetes prevention literature, the ways in which the *Inner and Outer Setting* constructs have been described use the standard CFIR broad definitions and lack specific operationalization required for quantitative measurement. To our knowledge, there are no National DPP specific studies that have used quantitative CFIR measures or evaluated these concepts across a large sample of delivery organizations.

**Fig. 1:**
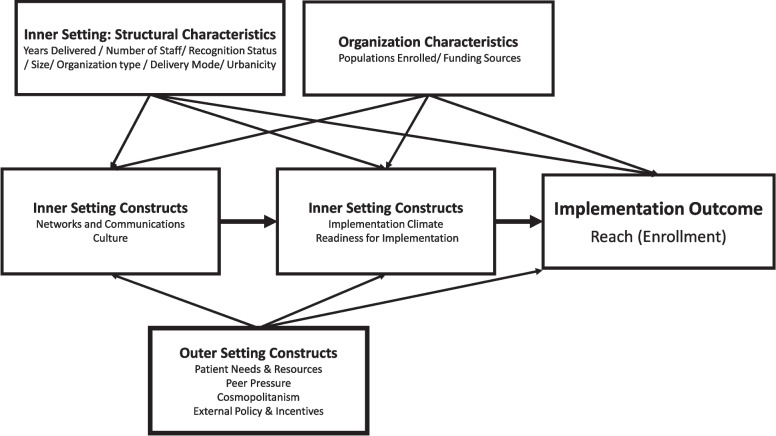
The hypothesized path model

The purpose of the study reported here was to quantitatively examine the relationships between CFIR *Inner* and *Outer Settin*g constructs and the implementation outcome of reach (Fig. 1). Using online survey data from National DPP implementers, our main research questions were [1] how do the *Inner* and *Outer Settin*g constructs impact reach (participant enrollment) for organizations implementing the National DPP lifestyle change program? and [2] in what ways do organizational characteristics such as organization type, size, location, etc., influence reach and these pathways directly or indirectly?

In addition, instead of simply understanding the link between CFIR constructs and implementation outcomes, we also aimed to explore the relationship between the *Inner* and *Outer Settin*g constructs. Particularly, we have emphasized the implementation focused constructs within the *Inner Setting: Implementation Climate* and *Readiness for Implementation* as key mediators. While the *Inner Setting* constructs *Networks and Communication* and *Culture* represent general organizational internal context, the two implementation focused ones are more focused on the internal context related to implementing the specific intervention. We think that the state of these general *Inner Setting* constructs may facilitate or hinder the implementation focused ones. Lastly, the *Inner Setting Structural Characteristics* and the *Outer Setting* constructs may impact the other *Inner Setting* constructs and implementation outcomes at multiple points in the model.

This rationale has led us to our three main hypotheses:

- H1. Inner Setting constructs (Networks and Communication and Culture) have an indirect relationship with the implementation outcome of reach mediated through implementation climate and readiness for implementation.
- H2. Outer Setting constructs (Patient Needs and Resources; Cosmopolitanism; Peer Pressure; External Policy and Incentives) may have direct and/or indirect relationships with the implementation outcome of reach as well as the Inner Setting constructs along the focal pathway.
- H3. Inner Setting Structural Characteristics and other organization characteristics (organization type, number of years implementing the program, region, urban/rural location, size, and recognition status) may have direct and/or indirect relationships with the implementation outcome of reach as well as the inner setting constructs along the focal pathway.

## Methods

This study was a part of a sequential, mixed-methods evaluation and involved a cross-sectional online survey (Qualtrics) conducted in August–September 2021 with National DPP staff. The National DPP is a year-long lifestyle change program delivered by National DPP organizations which includes 16hour- long sessions delivered over 6 months, followed by six additional sessions delivered over the subsequent 6 months [5]. The National DPP curriculum targets a number of health behavior constructs (self-efficacy, attitudes, knowledge, beliefs, social support, etc.) to change behavior and achieved the health outcome goals for participants (5–7% weight loss over 12 months and increased physical activity levels per week). National DPP staff members had one or more of the following roles: lifestyle coach, master trainer, and program coordinator. Lifestyle coaches deliver the program to participants. Master trainers are experienced lifestyle coaches that train lifestyle coaches within the same delivery organization. Program coordinators supervise daily operations of the program, provide guidance and support to the coaching staff, and monitor and submit all program data to the CDC.

National DPP staff were reached through Emory’s Diabetes Training and Technical Assistance Center (DTTAC), a CDC-recognized National DPP lifestyle coach and master trainer training program. This paper focuses on the analysis of the CFIR items, participant enrollment numbers (outcome of interest), and organization characteristics data. This study was reviewed and approved by Emory University’s Institutional Review Board (STUDYID00002611).

### Sampling and data collection

Study participants were recruited from DTTAC’s National DPP implementer population. Over the last 10 years, DTTAC has directly trained over 5000 lifestyle coaches representing over 2000 organizations across all 50 states. Using the most up to date list of DTTAC National DPP contacts, the online survey was distributed to 6470 email addresses in August 2021 to National DPP implementers who have participated in Emory’s DTTAC Lifestyle Coach training, DTTAC Master Trainer Select training, and/or subscribed to the center’s resources. All active National DPP program implementers who are either a lifestyle coach, master trainer, and/or program coordinator were eligible for the survey. Funding allowed for the first 336 respondents to receive a $15 Amazon gift card for their participation. The survey was active for 5 weeks. Weekly email reminders were sent to encourage participation. A total of 681 eligible responses were collected, and after data cleaning for completion 587 responses were included in the analysis.

### Instrument development and measures

The survey instrument included 101 items: 23 items requesting information about the respondent (their role, demographic info, etc.), their organization characteristics (type, location, length of delivery, etc.), and enrollment level to date; 38 Likert scale items related to the CFIR inner and outer setting constructs; and 40 items of the Program Sustainability Assessment Tool (see Table 1 and supplemental files for full survey instrument; the Program Sustainability Assessment Tool items were not used for the analysis described in this paper and are published elsewhere). Respondents were asked to report their organization’s total participant program enrollment to date. This enrollment number is our measure for reach, the outcome of interest for this analysis. Since there are no standard measures for all CFIR inner and outer setting constructs, items from existing scales and recent studies were examined for relevance and psychometric properties [19, 29, 30]. The selection of existing items and development of new items for the survey was heavily based on the preceding qualitative study examining these inner and outer setting constructs with 30 National DPP organization implementers [28]. The preliminary analysis of that qualitative study provided insight into which CFIR constructs and subconstructs were most relevant for this population and program. For example, the subconstructs, *Leadership Engagement* and *Available Resources*, within *Readiness for Implementation* were discussed heavily in the interviews, and therefore, multiple items were used for each of those constructs. Conversely, A*ccess to Knowledge and Information* and *Peer Pressure* items were not as emphasized and were removed to reduce the length of the survey.

To reduce the survey length further, the CFIR *Inner Setting* construct *Structural Characteristics* was operationalized using 19 of the organization characteristics items (e.g., length of delivery, number of staff, etc.) instead of Likert scale questions. This use of objective organization variables to measure *Structural Characteristics* has also been used in other CFIR studies [31, 32]. Organization characteristics variables regarding funding sources and populations enrolled were not included in structural characteristics as these are outside of the definition of the construct. All survey items were discussed and reviewed with the study team and pilot tested by subject matter experts at Emory’s DTTAC, who suggested wording changes and possible areas to reduce survey length.

The final survey included 38 CFIR items, 24 were adapted from existing scales and studies that have measured these specific CFIR constructs and subconstructs [19, 29, 30]. Adaptation of items primarily focused on tailoring language for the National DPP context, such as inserting the program name and terminology relevant to their implementation. Fourteen items were created based on insight from the qualitative study and subject matter expert input (Table 1). Respondents answered survey items based on their organization’s entire period of delivery of the National DPP.

### Data analyses

Data was exported from Qualtrics and analyzed using the SAS Software Version 9.4. and Mplus Version 8.3. Data were cleaned (screened, diagnosed, and edited for suspected data abnormalities) and missing data reviewed in accordance with standard data cleaning procedures [33]. Descriptive statistics were first performed, and all variables of interest were examined for normality and outliers. Upon review of variables, the outcome of interest, enrollment, was recalculated to remove four outliers above the 99th percentile. These outliers were large online delivery companies very different from the majority of the National DPP organizations in the sample. These outliers were removed to help normalize the data; in addition, this enrollment was scaled (divided by 100) to assist with comparison across other variables.

For all CFIR Likert scale items, scales were computed, and Cronbach’s alpha was used to assess the internal consistency of the scale for each of the CFIR constructs. Correlation matrices and Pearson coefficient were reviewed to understand the degree of overlap between related items within constructs. Items within each CFIR construct were averaged to create construct variables: 4 inner setting and 3 outer setting variables. Bi-variates and linear regression models were assessed to examine all CFIR and organization characteristic predictor variables related to the outcome variable (enrollment).

We tested our three hypotheses using structural equation modeling in Mplus. For the *Structural Characteristics*, 19 organizational characteristics variables were included in the model for the following: years of delivery, staffing, Diabetes Prevention Recognition Program (DPRP) recognition status,^1^ organization size, organization type, delivery mode, and urbanicity. Run separately from these *Structural Characteristics variables* were six other organization characteristics variables around populations enrolled and funding sources. To run the structural equation model, all variables were transformed into dichotomous (Yes/No) variables for each category. For example, each organization type, DPRP status level, organization size category was a separate variable. In addition, delivery mode variables were combined into like categories and reduced to “in-person” and “virtual” delivery variables. Due to the high number of respondent and organization characteristic variables and some small sample sizes within racial and ethnic populations served categories, populations enrolled also were combined and reduced to compare only organizations with only White participants enrolled and those with only non-White participants enrolled. Other participant categories were not used for this analysis. A total of 26 CFIR variables and 6 organization characteristics were included in the final model (Supplemental Table C2). Enrollment was the outcome of interest. Statistical significance for all tests was determined at the alpha = 0.05 level. Model fit was evaluated using standard goodness of fit indices criteria [34]. Structural equation modeling aims to test models and then interpret relationships between variables. Due to model complexity, multiple model fit indices are used to assess how well the model fits the data [35]. Lastly, model fit indices do not imply the relationships are strong or hypothesis correct but are a necessary condition before assessing significant relationships within the analysis.

**Table 2:** Respondent characteristics (*N* = 586)

	**Total Survey Respondents**	
**Respondent and organization characteristics**	***n***	**%**
**DPRP status**		
Full recognition	304	51.9%
Pending/preliminary	126	21.5%
None	52	8.9%
I do not know/missing	104	17.7%
	***n* (%)**	**Mean (SD)**
**Years delivered**	500 (85.3%)	4.51 (3.06)
**Enrollment to date**	357 (60.9%)	1758 (26,524.44)
**Enrollment scaled (divided by 100 + outliers removed)**	353 (60.2%)	1.83 (3.59)
**Lifestyle coaches at organization**	512 (87.4%)	7.1 (12.58)
**Non-lifestyle coach DPP staff**	500 (85.3%)	1.94 (7.78)
**Number of staff dedicated to national DPP 100%**	478 (81.6%)	1.94 (8.39)
**Organization type**		
Healthcare/hospitals	180	30.7%
Community-based healthcare	129	22.0%
Community-based organizations	55	9.4%
Government agencies	80	13.7%
Academic	43	7.3%
Health insurers, employers, other	91	15.5%
Missing	8	1.4%
**Organization size** (number of people served annually across all programs and services)		
Small (0–1000 people)	163	27.8%
Medium (1000–50,000 people)	163	27.8%
Large (over 50,000 people)	60	10.2%
I don’t know/missing	200	34.1%
**Delivery mode**		
In-person small group (meetings with up to 20 participants)	279	47.6%
In-person large group (meetings with 21 or more participants)	19	3.2%
In-person (small or large group)	279	47.6%
Distance (interacting live with all participants as a group using video and/or audio)	323	55.1%
Online (Using a platform for participants to engage with the content on their own—not a live group meeting)	98	16.7%
Hybrid (combination of modes)	142	24.2%
Virtual (distance, online, hybrid)	447	76.3%
Other	40	6.8%
**Location/urbanicity**		
Rural location	233	39.8%
Suburban location	190	32.4%
Urban location	237	40.4%
**Populations enrolled**		
White/Caucasian	361	61.6%
Black/African American	257	43.9%
Hispanic/Latino	177	30.2%
American Indian/Alaskan Native	67	11.4%
Asian	56	9.6%
Hawaiian Native/Pacific Islander	14	2.4%
Other	26	4.4%
Missing	65	11.1%
*Based on responses above:*		
Programs with only White participants enrolled	131	22.4%
Programs with only non-White participants enrolled	160	27.3%
**National DPP funded/supported by:**		
Federal government/CDC funding	112	19.1%
Medicare and/or Medicaid	68	11.6%
State or local government funding	114	19.5%
State employee coverage benefits	24	4.1%
Grant funding	195	33.3%
Missing	212	36.2%
**How would you describe your current DPP enrollment level?**		
We need to decrease our enrollment numbers (over capacity)	4	0.7%
We are comfortable at this level of enrollment	88	15.0%
We are actively working to increase our enrollment numbers	329	56.1%
We would like to increase our enrollment, but this is all we have capacity for at the moment	62	10.6%
Missing	103	17.6%
**To what extent do you agree or disagree that COVID-19 prohibited you from enrolling the desired number of participants into your program at this time?**		
Strongly disagree	46	7.8%
Disagree	63	10.8%
Neither agree nor disagree	91	15.5%
Agree	143	24.4%
Strongly agree	146	24.9%
Missing	97	16.6%
	***n* (%)**	**Mean (SD)**
Average score	489 (83.4%)	3.57 (1.29)
**Respondent role (may have more than 1)**	***n***	**%**
Lifestyle coach	538	91.8%
Program coordinator	222	37.9%
Master trainer	56	9.6%
**Respondent gender**		
Woman	395	67.4%
Man	36	6.1%
Other	2	0.3%
Missing	153	26.1%
**Respondent race/ethnicity**		
White/Caucasian	268	45.7%
Black/African American	76	13.0%
Hispanic/Latino	53	9.0%
American Indian/Alaskan Native	22	3.8%
Asian	16	2.7%
Other	4	0.7%
Hawaiian Native/Pacific Islander	2	0.3%
Missing	157	26.8%
**Respondent age range**		
Under 25 years	13	2.2%
25–34 years	89	15.2%
35–44 years	101	17.2%
45–54 years	94	16.0%
55–64 years	99	16.9%
65 years or older	36	6.1%
Missing	154	26.3%

## Results

### Descriptive statistics

Of the 586 survey respondents, the majority belonged to National DPP delivery organizations with full recognition in the DPRP (51.9%), while 21.5% of organizations were in the pending or preliminary phases of the program, and others reported their organization not being involved or did not know or respond to the question (Table 2). The average length of program delivery was 4.5 years (SD = 3.1). Our outcome, enrollment to date, was self-reported by 357 respondents (61%). The average enrollment was 1758 participants (SD = 26,524; range 0 to 500,000); with the 4 enrollment outliers removed, the average enrollment decreased to 183 participants (SD = 359; range 0 to 35,000).

The average number of lifestyle coaches at respondent organizations was 7.1 (SD = 12.6), with an average of 1.9 (SD = 7.8). National DPP staff in other roles (non-lifestyle coaches) and an average of 1.9 (SD = 8.4) National DPP staff dedicated 100% to the program. The most common types of respondent organizations were healthcare/hospitals (30.7%); community-based healthcare (community health centers, federally qualified health centers, Indian Health Service, etc., 22.0%); health insurers; employers and other (e.g., private businesses; 15.5%); and government agencies (13.7%). About half of the respondents reported their organization was offering the program in an in-person small group format (47.6%); however, as survey administration was conducted during the COVID-19 pandemic, the vast majority (75%) also were or exclusively offering programs in some type of virtual mode. Of those who reported their organization size, small (0–1000 people served annually) and medium (1000–50,000 people) organizations were equally represented at 27.8% and 10.2% were from larger organizations (50,000 + people).

Respondent organizations enrolled mostly White (61.6%), Black (43.9%), and Hispanic/Latino (30.2%) populations. There were 131 respondents (22.4%) from organizations where only White participants happened to be enrolled and 160 (27.3%) from organizations with only non-White participants enrolled. Respondent organizations were primarily funded/supported by grant funding (33.3%), state or local government funding (19.5%), and/or federal government/CDC funding (19.1%).

When asked “How would you describe your current DPP enrollment level?”, 56.1% reported that they are actively working to increase enrollment numbers, 15% said they were comfortable with the current level of enrollment, and 10.6% said they would like to increase their enrollment; however, they were limited by capacity at the moment. Respondents also were asked “To what extent do you agree or disagree that COVID-19 prohibited you from enrolling the desired number of participants into your program at this time?” About half of respondents (49.3%) said they agreed or strongly agreed with this statement, 15.5% neither agreed nor disagreed, 18.6% either disagreed or strongly disagreed, and 16.6% did not respond.

Respondents often had multiple National DPP roles, 91.8% said they were lifestyle coaches, 37% were program coordinators, and 9.6% master trainers. Respondents were mostly women (67.4%), White (45.7%), and fairly equally represented across age groups. Demographic questions were asked at the end, and 26% of missing responses were due to not finishing the survey.

### CFIR items

Each of the 38 CFIR items had between a 60 and 79% (351–463 responses) response rate (Table 3). Items were rated on a 1–5 bi-polar scale. Higher ratings indicate agreement with positive statements related to the implementation construct, and there was a 3.82 average rating across all items. The items with the highest and lowest average ratings were both in the Implementation Climate construct. Cronbach’s alpha ranged from 0.69 (external policies and incentives) to 0.93 (leadership engagement) (Table 4).

**Table 3:** CFIR inner and outer setting Likert scale items and mean scores

CFIR item	Question (response scale strongly disagree 1 to strongly agree 5)	*N*	Mean	Std
Networks and Communication	We have regular project meetings with our organization’s National DPP team members/staff	429	3.76	1.2
Networks and Communication	There is regular involvement of staff in National DPP planning and implementation	435	3.78	1.1
Networks and Communication	We provide regular feedback to organization management on progress of program activities and resource needs	434	3.95	1.0
Networks and Communication	We provide regular feedback to organization staff on effects of the National DPP on participant outcomes	428	3.92	1.0
Networks and Communication	We consistently use an internal referral processes (referrals within your organization to the program) for the National DPP	417	3.86	1.1
Culture	People at all levels openly talk about what is and isn’t working	448	3.95	1.0
Culture	We regularly take time to reflect on how we do things	452	4.01	0.9
Culture	People in this organization operate as a real team	451	4.03	1.0
Culture	The National DPP aligns well with the mission and/or vision at our organization	463	4.33	0.8
Implementation Climate	Our organization has established National DPP goals that the program staff are expected to help meet (i.e., increase DPP enrollment rates)	421	3.78	1.0
Implementation Climate	Organization National DPP staff have the support they need to implement the National DPP	442	3.83	1.0
Implementation Climate	Organization National DPP staff receive acknowledgement (i.e., bonus, awards, public acknowledgement, etc.) for implementing the National DPP successfully	406	2.94	1.2
Implementation Climate	The National DPP is a top priority of the organization	440	3.30	1.1
Implementation Climate	The National DPP fits well with our organization’s existing workflow and systems	442	3.85	0.9
Implementation Climate	There is a strong need for this program at our organization	459	4.34	0.8
Leadership Engagement	Organization leadership makes sure that staff have the time necessary to implement the National DPP	445	3.86	1.0
Leadership Engagement	Organization leadership makes sure that staff have the space (physical for in-person classes and/or a virtual/online platform) necessary to implement the National DPP	448	3.95	0.9
Leadership Engagement	Leadership in this organization create an environment where things can be accomplished for the National DPP	450	3.90	0.9
Leadership Engagement	Organization leadership promotes an environment that is an enjoyable place to work on the National DPP	446	3.95	0.9
Leadership Engagement	Leadership strongly supports the National DPP implementation efforts	450	3.92	1.0
Available Resources	Financial resources to support the implementation of the National DPP	426	3.64	1.1
Available Resources	Number of staff (lifestyle coaches and others) to support the implementation of the National DPP	445	3.71	1.1
Available Resources	Basic staff training to facilitate the implementation of the National DPP	456	4.02	0.9
Available Resources	Equipment/materials to facilitate the implementation of the National DPP	456	4.06	0.9
Available Resources	Facilities/space to host the National DPP in-person	432	3.94	0.9
Available Resources	Virtual/Distance/Online platform to host the National DPP via distance or online delivery	446	4.03	1.0
Patient Needs and Resources	Our organization does a good job of assessing participant needs and barriers to enrolling in the National DPP	440	3.86	0.9
Patient Needs and Resources	Our organization uses data from participants to improve program delivery	423	3.95	0.9
Patient Needs and Resources	Our organization uses data from participants to improve recruitment and enrollment strategies	425	3.87	0.9
Patient Needs and Resources	Our organization has taken steps to reduce barriers to enrollment for participants	433	3.92	0.9
Patient Needs and Resources	There is high demand for the National DPP lifestyle change program in the geographic region our organization serves	432	3.69	1.1
Cosmopolitanism	Our organization/staff engages in inter-organizational networking or partnerships (coalitions, meetings, conferences, group trainings, etc.) related to diabetes, prediabetes, and/or the National DPP	421	3.91	0.9
Cosmopolitanism	Our external/community partners promote our National DPP lifestyle change program	406	3.70	1.0
Cosmopolitanism	Our program has an effective participant referral processes with external organizations (healthcare providers, community partners, other National DPP organizations, etc.) in place	416	3.40	1.1
Cosmopolitanism	Our organization works collaboratively with other organizations who deliver the National DPP (i.e., inter-organization referrals, marketing, resource sharing, etc.)	404	3.50	1.1
External Policies and Incentives	Our organization receives acknowledgement for using an evidence-based program	390	3.73	1.0
External Policies and Incentives	External funding for diabetes prevention supports our organization’s implementation of the National DPP	351	3.56	1.2
External Policies and Incentives	The CDC DPRP reporting requirements are helpful for our organization’s implementation of the National DPP	402	3.56	1.1

**Table 4:** CFIR Likert scale construct scores and Cronbach’s alpha

CFIR construct aggregated mean scores	*N*	Mean	Std	Number of items	Cronbach’s alpha
*Inner Setting*					
Networks and communication	451	3.85	0.9	5	0.89
Culture	469	4.08	0.8	4	0.86
Implementation climate	467	3.69	0.8	6	0.84
Leadership engagement	459	3.91	0.8	5	0.93
Available resources	463	3.91	0.7	6	0.86
*Outer Setting*					
Patient needs and resources	457	3.87	0.8	5	0.87
Cosmopolitanism	444	3.63	0.8	4	0.81
External policies and incentives	433	3.64	0.9	3	0.69

### Structural model

**Fig. 2:**
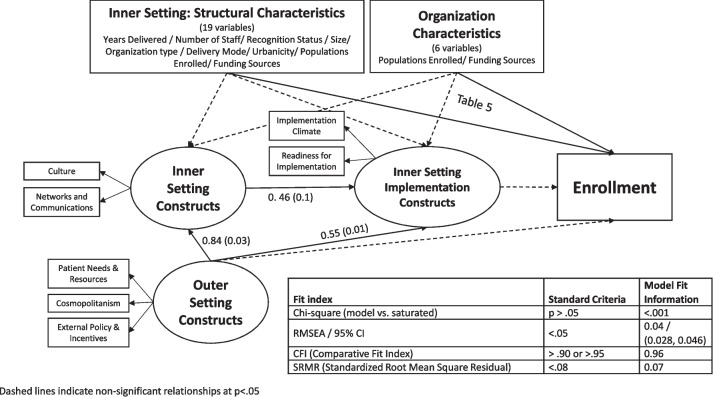
Final structural equation model (*n* = 445)

The initial hypothesized model (Fig. 1) did not explain the data well (had poor model fit). Due to the high correlation between items, latent variables were created to capture the two-domains of inner setting constructs (culture and networks and communication; implementation climate and readiness for implementation) and the outer setting constructs (patient needs and resources, cosmopolitanism, and external policy and incentives) in our model (Fig. 2, Supplemental Table A1). Latent variables are measured through indicator variables and modeled as caused by those indicators [34]. This change in model structure allowed us to remain consistent to our theoretical understanding of the relationships in our operationalized path model. The final model fit the data well (Fig. 2).

In the final model, there was a significant direct path from *Inner Setting Constructs* to the *Inner Setting Implementation Construct*, as well as significant direct paths from the *Outer Setting Constructs* to both *Inner Setting* latent variables. None of the latent *Inner* and *Outer Setting* variables were directly or indirectly associated with enrollment. Instead, seven of the *Structural Characteristics* variables (length of delivery, number of lifestyle coaches, number of full-time staff, large organization size, and organizations delivering in rural, suburban, and/or urban settings) had significant positive direct relationships with enrollment (Table 5). For example, these parameters can be interpreted as for every additional lifestyle coach at an organization, enrollment increases by 47 participants. One of the *Structural Characteristics* variables, “academic type organizations”, and one of the organization characteristics variables “organizations with only non-White participants enrolled in their National DPP lifestyle change programs”, had significant negative direct relationships with enrollment. Results for all variables are included in Supplemental Table C.

**Table 5:** Standardized significant coefficients (*n* = 445)

**Outcome**		**Coefficient (SE)**	***p*-value**
	**Structural characteristic variable**		
Enrollment (scaled/100)	Years delivered	0.28 (0.05)	< .001
	Number of lifestyle coaches at organization	0.47 (0.06)	< .001
	Number of staff dedicated to National DPP 100%	0.34 (0.12)	0.004
	Organization size: large (over 50,000)	0.14 (0.05)	0.005
	Organization type: academic	− 0.13 (0.04)	0.001
	Location: rural	0.21 (0.06)	< .001
	Location: suburban	0.16 (0.06)	0.009
	Location: urban	0.16 (0.07)	0.015
	**Organization characteristic variable**		
	Programs with only non-White participants enrolled	− 0.10 (0.05)	0.030

R-squared on enrollment = 0.64

## Discussion

This study aimed to identify CFIR *Inner* and *Outer Setting* measures to explore relationships between internal and external organization factors, organizational characteristics, and participant enrollment. Of our three hypotheses, only the last one (H3), was found to be partially supported; the *Inner Setting Structural Characteristics*, operationalized by 19 organization characteristics, such as length of delivery, staff size, and organization type, had significant direct relationships with participant reach. In addition, one organization characteristic variable (programs with only non-White participants enrolled) also had a significant direct relationship with participant reach.

Our CFIR Likert scale items had good internal consistency and provide insight into implementation areas of strength and weakness. For example, two *Implementation Climate* items had the lowest average ratings; the first identifies a lack of staff acknowledgements (bonuses, awards, public recognition, etc.) for implementing the program. The second lowest rated CFIR item indicated that the respondents on average are neutral (did not agree or disagree) about if the National DPP is a top priority at their organization. The highest rated items revealed that respondents believed there was a strong need for the program (*Implementation Climate-Relative Priority*) and that the program was aligned well with the organization values and mission (*Culture*). This reveals that there may be a disconnect between the National DPP staff who completed this survey and their organization leadership and that perhaps more buy-in from leadership to make this program a higher priority and providing incentives to support staff in their work may be areas for growth.

The length of delivery, number of lifestyle coaches, number of full-time staff, large organization size, and organizations delivering in rural, suburban, and/or urban settings all had positive significant direct relationships with enrollment. Many of these variables (e.g., staff size and organization size) reflect organization capacity which is a critical piece of effective program delivery [36]. Longer length of delivery for example indicates an organization has had the capacity to deliver the program over time and therefore more opportunities to reach more participants. Other variables point to the importance of setting, all locations rural, suburban, and urban were positively related to enrollment. Rural settings had a slightly higher effect size (0.21 vs 0.16 in suburban and urban settings). This finding is interesting given the other literature which has found recruitment to be a challenge in rural areas [37, 38]. This may be due to the relatively high representation of respondents delivering the program in rural communities in our sample.

Moreover, academic organizations and organizations with only non-White participants enrolled in their National DPP lifestyle change programs had negative direct relationships with enrollment. Based on other National DPP studies, we know that White participants are more likely to be enrolled in the program, indicating that non-White populations may be harder to reach and recruit [3]. It is unclear why academic organizations in particular are negatively associated with enrollment. It may be that these institutions run only a few cohorts for their academic community that eligible participants are uncomfortable enrolling in a program like this at work and/or do not do outreach within the broader community. More examination of organization types and their ability to reach participants should be explored in future work. However, the lack of findings among organization types does support the CDC’s National DPP vision that this program can be implemented in various settings [39].

This survey data also supports “reach” as an important programmatic goal for National DPP implementers. The majority reported they are actively working to increase enrollment numbers, and about the same proportion of respondents also reported that the COVID-19 pandemic has impacted their enrollment negatively. Research on the challenges and adaptations of program delivery during COVID-19 have started to appear in the published literature and reflect similar findings around reach [40]. Typically, reach literature has focused on participant characteristics and recruitment method predictors to enrollment/participation, not organizational level characteristics [18]. In 2019, the CDC published findings from an evaluation examining implementation across 164 of National DPP organizations using the RE-AIM framework [41]. They identified recruitment strategies associated with higher overall attendance and longer participation duration included using self-referral or word of mouth, providing non-monetary incentives for participation, and using cultural adaptations to address participants’ needs. However, they did not report any findings on any other context-related predictors of enrollment at delivery sites.

Findings from this study are similar to other evaluation findings and research of the National DPP. For example, regarding staffing, the initiative has promoted building up the DPP workforce through many methods (e.g., standardized training, resource centers, state and federal technical assistance) and been largely successful in scaling the number of delivery sites across the country (Ackermann & O’Brien, 2020). However, evaluations of the program at specific sites often indicate more outreach, involvement, and communication with participants and their referring healthcare providers by National DPP staff would be helpful to increase recruitment and retention in the program [41, 42]. Data from our sample of implementing staff showed that on average organizations have less than 2 staff who are dedicated to working on the National DPP full time. This may not be sufficient for a program as resource intensive as the National DPP and for program sustainability. More research should be conducted in this area to understand staff capacity needs that go beyond adequate training and ask questions about time and resources needed to meet enrollment and other programmatic goals. Other characteristics associated with enrollment in our findings may not be modifiable like staffing (e.g., organization type) but can provide insight for strategic outreach to new organizations who might be best suited to adopt and deliver the program.

Lastly, as described by our findings and other studies, outreach to rural and non-White participants should be considered priority areas for enrollment growth [2, 22, 37, 38, 43]. In a 2019 published analysis of the National DPP, although diabetes prevention interventions are a high need in rural areas, there were significantly fewer rural counties with access to a National DPP site compared with urban counties (14.6% vs. 48.4%, respectively, *p* < 0.001) [37]. The authors recommended identifying alternative dissemination strategies that address the unique barriers to implementation faced by rural communities to increase program access. Emory’s DTTAC has focused heavily on providing National DPP support to rural areas, which may be why representation among respondents providing the program in rural locations was high (40%) and rural delivery was positively related to enrollment. Adaptations of the DPP in rural communities have been evaluated and encourage use of telehealth/virtual technology to provide the program, as well as partnerships with local, accessible resources (e.g., recreational space at local institutions) to support behavior changes [38]. There have also been a number of adaptations and strategies to address the disparities in reach regarding racial and ethnic minorities and men as well [2].

### Strengths and limitations

Strengths of this study include the use of adapted and newly developed quantitative CFIR measures and the use of structural equation modeling to understand relationships between these factors and participant enrollment (reach). Our recruitment was limited by Emory’s DTTAC contact list, and there may be differences between this group and the larger National DPP population of implementers, for example the large representation from organizations delivering in rural communities. However, we still were able to capture data from a relatively large and diverse sample of respondents.

Another limitation was that we had to rely on respondent summitted enrollment data and were not able to verify this with program records. In addition, around 40% of respondents did not know the number of participants they had enrolled to date, which also indicates a lack of awareness of staff on this important metric of program implementation. We were also unable to accurately assess if multiple people from same organization completed the survey due inconsistencies in how organization names were provided by respondents and could not account for this clustering in our analyses. Further research should examine how perspectives on program implementation and CFIR item ratings vary between staff within the same organization.

Our analysis only showed the *Inner Setting Structural Characteristics* to have a direct relationship with enrollment. One reason for this may be due to the fact that organization characteristics variables could also be considered part of other CFIR constructs. For example, the sources of funding/support variables overlap with aspects of the *External Policies and Incentives* construct. This highlights one of the challenges of measuring CFIR constructs quantitatively as their definitions are so multifaceted [8, 44]. In addition, to reduce participant burden we shortened the length of the survey removing additional CFIR items which may have increased comprehensiveness of these constructs. While our study focuses only on the *Inner* and *Outer Setting*, this may have still been too much and future studies may want to focus on specific constructs. We also only used cross-sectional data to explore these possible relationships in our path model; to more accurately understand mediation among these constructs, future studies should collect prospective data at multiple time points.

Furthermore, CFIR 2.0 has been recently released and provides new aspects and/or segmentation of constructs to help address these issues [45]. For example, currently, the *Outer Setting* does not describe differences in participant populations, but we know from our findings that location setting (rural, suburban, urban) and race/ethnicity also are important factors that are part of the external context in which an intervention is implemented. As CFIR measures are still in development and testing in the field, it will require many more research applications like this to understand the most effective way to capture each of these domains, constructs, and sub-constructs. In the future, to further test these measures, we hope to examine other implementation outcomes, such as extent of implementation of DPP, the quality of implementation, and/or sustainability.

Related to our analysis, latent variables usually recommend at least three items per latent construct; however, we only had two variable indicators for the inner setting latent variables [34]. We did find good internal consistency for all of our CFIR constructs, and this is also strengthened by our mixed methods study design building off of a previous qualitative study to inform items selection, adaptation, and creation in this quantitative study [28]. We also found good model fit with our latent variables with the divided the *Inner Setting* constructs and found significant paths between all three latent CFIR variables. This is also a new exploration from other applications of CFIR and future research may want to continue to examine relationships among constructs in addition to CFIR constructs on implementation outcomes. Another limitation was the consolidation of many organizational characteristics (e.g., reducing populations served to White or non-White). Future studies may want to focus more on particular organizational characteristics more specifically. To our knowledge, this is one of the first applications of quantitative CFIR items using structural equation modeling, which we believe helps expand the possibilities of CFIR and implementation science measures and methods.

## Conclusions

This study provides valuable insight into the internal and external organizational factors related to National DPP enrollment. Our findings suggest that to facilitate enrollment, program implementers should prioritize the *Structural Characteristics* and other organization characteristics such as adequate staffing, expanding the program in multiple locations (rural, suburban, and urban), and improving recruitment of non-White participants. Participant reach (enrollment) is an important implementation outcome for the National DPP and vital to making population-level decreases in diabetes incidence in the USA. While the CFIR latent variables were not significantly related directly or indirectly to enrollment, the item responses and construct scores provide useful information regarding implementation strengths and areas for implementation support. Implementing staff believe that the National DPP is a needed program and aligned with their organization’s mission. Those working to scale the National DPP should ensure the program is a priority for organization leadership and all the necessary staff motivators and supports are in place. In addition, other health prevention programs can use the methods and findings from this study to further understand and inform the impact of organization factors on implementation outcomes.

### Supplementary Information

**Additional file 1: Table A1.** CFIR Constructs Correlation Matrix.

**Additional file 2: Table A2.** Organization Variables Correlation Matrix.

**Additional file 3: Table B.** Organization Characteristics and CFIR Item Bi-variates.

**Additional file 4: Tables C1-3.** Structural Equation Model Results.

## Data Availability

Quantitative data is available by reasonable request to the corresponding author.

## Abbreviations

CDC: Centers for Disease Control and Prevention
CFIR: Consolidated Framework for Implementation Research
DPP: Diabetes Prevention Program
DPRP: Diabetes Prevention Recognition Program
DTTAC: Diabetes Training and Technical Assistance Center

## Footnotes

1 The DPRP provides national quality standards to ensure organizations are delivering the program with fidelity. These standards and procedures are updated every 3 years based on new dietary, physical activity, self-efficacy, delivery modality, and other type 2 diabetes prevention evidence. CDC-recognized organizations work toward progressing from pending to preliminary to full recognition status.
