# Supplementary material for: A structural equation model of CFIR inner and outer setting constructs, organization characteristics, and national DPP enrollment
Source: Implement Sci Commun. 2023 Nov 17;4:142. doi: 10.1186/s43058-023-00522-3 (PMC10657127; doi:10.1186/s43058-023-00522-3)
Supplement: Supplementary file 1 — Additional file 1: Table A1. CFIR Constructs Correlation Matrix. [file 43058_2023_522_MOESM1_ESM.docx]

**Table A1. CFIR Constructs Correlation Matrix**

|  | Networks and Communication | Culture | Implementation Climate | Leadership Engagement | Available Resources | Patient Needs and Resources | Cosmopolitanism | External Policies and Incentives |
| --- | --- | --- | --- | --- | --- | --- | --- | --- |
| Networks and Communication | 1 | 0.68665 | 0.65683 | 0.63404 | 0.54104 | 0.60149 | 0.48122 | 0.4684 |
|  |  | <.0001 | <.0001 | <.0001 | <.0001 | <.0001 | <.0001 | <.0001 |
|  | 451 | 449 | 446 | 442 | 443 | 437 | 427 | 421 |
| Culture | 0.68665 | 1 | 0.67439 | 0.71508 | 0.58656 | 0.61598 | 0.44942 | 0.44884 |
|  | <.0001 |  | <.0001 | <.0001 | <.0001 | <.0001 | <.0001 | <.0001 |
|  | 449 | 469 | 465 | 457 | 461 | 453 | 443 | 433 |
| Implementation Climate | 0.65683 | 0.67439 | 1 | 0.7552 | 0.65642 | 0.66144 | 0.57902 | 0.5963 |
|  | <.0001 | <.0001 |  | <.0001 | <.0001 | <.0001 | <.0001 | <.0001 |
|  | 446 | 465 | 467 | 457 | 461 | 454 | 443 | 432 |
| Leadership Engagement | 0.63404 | 0.71508 | 0.7552 | 1 | 0.71945 | 0.624 | 0.52601 | 0.53676 |
|  | <.0001 | <.0001 | <.0001 |  | <.0001 | <.0001 | <.0001 | <.0001 |
|  | 442 | 457 | 457 | 459 | 457 | 450 | 438 | 430 |
| Available Resources | 0.54104 | 0.58656 | 0.65642 | 0.71945 | 1 | 0.59105 | 0.51806 | 0.54359 |
|  | <.0001 | <.0001 | <.0001 | <.0001 |  | <.0001 | <.0001 | <.0001 |
|  | 443 | 461 | 461 | 457 | 463 | 454 | 442 | 432 |
| Patient Needs and Resources | 0.60149 | 0.61598 | 0.66144 | 0.624 | 0.59105 | 1 | 0.54124 | 0.56406 |
|  | <.0001 | <.0001 | <.0001 | <.0001 | <.0001 |  | <.0001 | <.0001 |
|  | 437 | 453 | 454 | 450 | 454 | 457 | 441 | 430 |
| Cosmopolitanism | 0.48122 | 0.44942 | 0.57902 | 0.52601 | 0.51806 | 0.54124 | 1 | 0.53702 |
|  | <.0001 | <.0001 | <.0001 | <.0001 | <.0001 | <.0001 |  | <.0001 |
|  | 427 | 443 | 443 | 438 | 442 | 441 | 444 | 425 |
| External Policies and Incentives | 0.4684 | 0.44884 | 0.5963 | 0.53676 | 0.54359 | 0.56406 | 0.53702 | 1 |
|  | <.0001 | <.0001 | <.0001 | <.0001 | <.0001 | <.0001 | <.0001 |  |
|  | 421 | 433 | 432 | 430 | 432 | 430 | 425 | 433 |
