# Supplementary material for: A structural equation model of CFIR inner and outer setting constructs, organization characteristics, and national DPP enrollment
Source: Implement Sci Commun. 2023 Nov 17;4:142. doi: 10.1186/s43058-023-00522-3 (PMC10657127; doi:10.1186/s43058-023-00522-3)
Supplement: Supplementary file 2 — Additional file 2: Table A2. Organization Variables Correlation Matrix. [file 43058_2023_522_MOESM2_ESM.docx]

**Table A2. Organization Variables Correlation Matrix**

|  | DPRP Status | Years of Program Delivery | Number of Lifestyle Coaches | Number of Full Time National DPP Staff | Organization Size | Org Type: Healthcare/Hospitals | Org Type: Community-based healthcare | Org Type: Community-based organizations | Org Type: Government agencies | Org Type: Academic | Org Type: Health insurers, Employers, Other | Delivery Mode: In Person (small or large group) | Delivery Mode: Distance, Online, and/or Hybrid | Rural Location | Suburban Location | Urban Location | White Participants Only | Non-white Participants Only | Funding Source: Federal Government/ CDC Funding | Funding Source: Medicaid and/or Medicare | Funding Source: State or Local Government Funding and/or State Employee Benefits | Funding Source: Grant funding |
| --- | --- | --- | --- | --- | --- | --- | --- | --- | --- | --- | --- | --- | --- | --- | --- | --- | --- | --- | --- | --- | --- | --- |
| DPRP Status | 1.00 | 0.30 | 0.11 | 0.13 | 0.10 | 0.13 | -0.04 | 0.08 | -0.09 | 0.00 | -0.08 | -0.02 | 0.26 | -0.09 | 0.11 | 0.02 | 0.11 | -0.16 | 0.04 | 0.14 | 0.04 | -0.02 |
|  |  | <.0001 | 0.03 | 0.01 | 0.05 | 0.01 | 0.38 | 0.08 | 0.04 | 0.95 | 0.06 | 0.73 | <.0001 | 0.06 | 0.01 | 0.64 | 0.02 | 0.00 | 0.38 | 0.00 | 0.42 | 0.64 |
|  | 482 | 427 | 439 | 413 | 416 | 482 | 482 | 482 | 482 | 482 | 482 | 482 | 482 | 482 | 482 | 482 | 482 | 482 | 482 | 482 | 482 | 482 |
| Years of Program Delivery | 0.30 | 1.00 | 0.17 | 0.11 | 0.09 | -0.01 | 0.04 | -0.05 | 0.00 | 0.04 | -0.03 | 0.03 | 0.02 | 0.01 | 0.03 | 0.02 | 0.07 | -0.03 | 0.06 | 0.09 | 0.12 | 0.11 |
|  | <.0001 |  | 0.00 | 0.02 | 0.06 | 0.91 | 0.40 | 0.31 | 0.93 | 0.36 | 0.57 | 0.44 | 0.74 | 0.81 | 0.52 | 0.60 | 0.12 | 0.45 | 0.15 | 0.05 | 0.01 | 0.02 |
|  | 427 | 500 | 489 | 457 | 466 | 500 | 500 | 500 | 500 | 500 | 500 | 500 | 500 | 500 | 500 | 500 | 500 | 500 | 500 | 500 | 500 | 500 |
| Number of Lifestyle Coaches | 0.11 | 0.17 | 1.00 | 0.23 | 0.14 | 0.00 | -0.06 | 0.02 | -0.06 | 0.06 | 0.07 | -0.05 | 0.08 | -0.11 | 0.09 | 0.15 | -0.06 | -0.03 | 0.08 | 0.05 | 0.12 | 0.01 |
|  | 0.03 | 0.00 |  | <.0001 | 0.00 | 0.95 | 0.18 | 0.72 | 0.21 | 0.18 | 0.13 | 0.30 | 0.07 | 0.01 | 0.04 | 0.00 | 0.20 | 0.51 | 0.07 | 0.24 | 0.01 | 0.80 |
|  | 439 | 489 | 512 | 473 | 478 | 512 | 512 | 512 | 512 | 512 | 512 | 512 | 512 | 512 | 512 | 512 | 512 | 512 | 512 | 512 | 512 | 512 |
| Number of Full Time National DPP Staff | 0.13 | 0.11 | 0.23 | 1.00 | 0.12 | -0.04 | -0.05 | 0.11 | -0.08 | -0.04 | 0.14 | -0.13 | 0.08 | -0.06 | -0.03 | 0.05 | -0.07 | 0.04 | 0.13 | 0.02 | 0.01 | -0.02 |
|  | 0.01 | 0.02 | <.0001 |  | 0.01 | 0.34 | 0.24 | 0.02 | 0.09 | 0.33 | 0.00 | 0.00 | 0.08 | 0.22 | 0.58 | 0.29 | 0.11 | 0.37 | 0.00 | 0.67 | 0.83 | 0.68 |
|  | 413 | 457 | 473 | 478 | 474 | 478 | 478 | 478 | 478 | 478 | 478 | 478 | 478 | 478 | 478 | 478 | 478 | 478 | 478 | 478 | 478 | 478 |
| Organization Size | 0.10 | 0.09 | 0.14 | 0.12 | 1.00 | 0.04 | -0.05 | -0.07 | 0.07 | 0.07 | -0.06 | -0.02 | -0.02 | -0.01 | 0.06 | 0.07 | -0.01 | -0.08 | -0.03 | -0.05 | 0.00 | -0.03 |
|  | 0.05 | 0.06 | 0.00 | 0.01 |  | 0.33 | 0.29 | 0.13 | 0.14 | 0.10 | 0.20 | 0.59 | 0.60 | 0.76 | 0.20 | 0.11 | 0.77 | 0.10 | 0.57 | 0.26 | 0.95 | 0.58 |
|  | 416 | 466 | 478 | 474 | 490 | 490 | 490 | 490 | 490 | 490 | 490 | 490 | 490 | 490 | 490 | 490 | 490 | 490 | 490 | 490 | 490 | 490 |
| Org Type: Healthcare/Hospitals | 0.13 | -0.01 | 0.00 | -0.04 | 0.04 | 1.00 | -0.35 | -0.21 | -0.26 | -0.19 | -0.29 | 0.03 | 0.02 | -0.04 | 0.04 | -0.04 | 0.17 | -0.23 | -0.11 | 0.11 | -0.13 | -0.09 |
|  | 0.01 | 0.91 | 0.95 | 0.34 | 0.33 |  | <.0001 | <.0001 | <.0001 | <.0001 | <.0001 | 0.44 | 0.57 | 0.31 | 0.38 | 0.38 | <.0001 | <.0001 | 0.01 | 0.01 | 0.00 | 0.04 |
|  | 482 | 500 | 512 | 478 | 490 | 586 | 586 | 586 | 586 | 586 | 586 | 586 | 586 | 586 | 586 | 586 | 586 | 586 | 586 | 586 | 586 | 586 |
| Org Type: Community-based healthcare | -0.04 | 0.04 | -0.06 | -0.05 | -0.05 | -0.35 | 1.00 | -0.17 | -0.21 | -0.15 | -0.23 | 0.05 | -0.02 | 0.15 | -0.16 | -0.04 | -0.14 | 0.32 | 0.13 | -0.04 | -0.03 | 0.11 |
|  | 0.38 | 0.40 | 0.18 | 0.24 | 0.29 | <.0001 |  | <.0001 | <.0001 | 0.00 | <.0001 | 0.27 | 0.57 | 0.00 | 0.00 | 0.40 | 0.00 | <.0001 | 0.00 | 0.36 | 0.44 | 0.01 |
|  | 482 | 500 | 512 | 478 | 490 | 586 | 586 | 586 | 586 | 586 | 586 | 586 | 586 | 586 | 586 | 586 | 586 | 586 | 586 | 586 | 586 | 586 |
| Org Type: Community-based organizations | 0.08 | -0.05 | 0.02 | 0.11 | -0.07 | -0.21 | -0.17 | 1.00 | -0.13 | -0.09 | -0.14 | 0.02 | 0.01 | -0.03 | 0.06 | 0.02 | 0.02 | 0.10 | 0.02 | 0.07 | 0.03 | 0.06 |
|  | 0.08 | 0.31 | 0.72 | 0.02 | 0.13 | <.0001 | <.0001 |  | 0.00 | 0.03 | 0.00 | 0.61 | 0.73 | 0.41 | 0.12 | 0.61 | 0.56 | 0.01 | 0.59 | 0.11 | 0.50 | 0.16 |
|  | 482 | 500 | 512 | 478 | 490 | 586 | 586 | 586 | 586 | 586 | 586 | 586 | 586 | 586 | 586 | 586 | 586 | 586 | 586 | 586 | 586 | 586 |
| Org Type: Government agencies | -0.09 | 0.00 | -0.06 | -0.08 | 0.07 | -0.26 | -0.21 | -0.13 | 1.00 | -0.11 | -0.17 | -0.03 | -0.04 | 0.04 | 0.05 | -0.06 | -0.02 | -0.04 | 0.03 | -0.10 | 0.26 | -0.04 |
|  | 0.04 | 0.93 | 0.21 | 0.09 | 0.14 | <.0001 | <.0001 | 0.00 |  | 0.01 | <.0001 | 0.46 | 0.39 | 0.30 | 0.19 | 0.12 | 0.59 | 0.30 | 0.41 | 0.02 | <.0001 | 0.36 |
|  | 482 | 500 | 512 | 478 | 490 | 586 | 586 | 586 | 586 | 586 | 586 | 586 | 586 | 586 | 586 | 586 | 586 | 586 | 586 | 586 | 586 | 586 |
| Org Type: Academic | 0.00 | 0.04 | 0.06 | -0.04 | 0.07 | -0.19 | -0.15 | -0.09 | -0.11 | 1.00 | -0.12 | 0.07 | 0.08 | -0.01 | 0.01 | 0.05 | 0.02 | -0.17 | -0.02 | -0.02 | 0.06 | 0.02 |
|  | 0.95 | 0.36 | 0.18 | 0.33 | 0.10 | <.0001 | 0.00 | 0.03 | 0.01 |  | 0.00 | 0.08 | 0.05 | 0.72 | 0.72 | 0.24 | 0.60 | <.0001 | 0.62 | 0.63 | 0.17 | 0.57 |
|  | 482 | 500 | 512 | 478 | 490 | 586 | 586 | 586 | 586 | 586 | 586 | 586 | 586 | 586 | 586 | 586 | 586 | 586 | 586 | 586 | 586 | 586 |
| Org Type: Health insurers, Employers, Other | -0.08 | -0.03 | 0.07 | 0.14 | -0.06 | -0.29 | -0.23 | -0.14 | -0.17 | -0.12 | 1.00 | -0.10 | 0.03 | -0.09 | 0.05 | 0.13 | -0.05 | 0.02 | -0.03 | -0.02 | -0.09 | -0.01 |
|  | 0.06 | 0.57 | 0.13 | 0.00 | 0.20 | <.0001 | <.0001 | 0.00 | <.0001 | 0.00 |  | 0.02 | 0.49 | 0.03 | 0.27 | 0.00 | 0.24 | 0.58 | 0.49 | 0.58 | 0.03 | 0.76 |
|  | 482 | 500 | 512 | 478 | 490 | 586 | 586 | 586 | 586 | 586 | 586 | 586 | 586 | 586 | 586 | 586 | 586 | 586 | 586 | 586 | 586 | 586 |
| Delivery Mode: In Person (small or large group) | -0.02 | 0.03 | -0.05 | -0.13 | -0.02 | 0.03 | 0.05 | 0.02 | -0.03 | 0.07 | -0.10 | 1.00 | -0.22 | 0.09 | 0.02 | -0.08 | 0.11 | -0.10 | -0.01 | 0.05 | 0.02 | 0.04 |
|  | 0.73 | 0.44 | 0.30 | 0.00 | 0.59 | 0.44 | 0.27 | 0.61 | 0.46 | 0.08 | 0.02 |  | <.0001 | 0.03 | 0.65 | 0.07 | 0.01 | 0.01 | 0.78 | 0.23 | 0.68 | 0.28 |
|  | 482 | 500 | 512 | 478 | 490 | 586 | 586 | 586 | 586 | 586 | 586 | 586 | 586 | 586 | 586 | 586 | 586 | 586 | 586 | 586 | 586 | 586 |
| Delivery Mode: Distance, Online, and/or Hybrid | 0.26 | 0.02 | 0.08 | 0.08 | -0.02 | 0.02 | -0.02 | 0.01 | -0.04 | 0.08 | 0.03 | -0.22 | 1.00 | -0.10 | 0.08 | 0.12 | -0.06 | 0.01 | 0.07 | 0.09 | 0.04 | 0.07 |
|  | <.0001 | 0.74 | 0.07 | 0.08 | 0.60 | 0.57 | 0.57 | 0.73 | 0.39 | 0.05 | 0.49 | <.0001 |  | 0.02 | 0.06 | 0.00 | 0.17 | 0.84 | 0.11 | 0.03 | 0.31 | 0.09 |
|  | 482 | 500 | 512 | 478 | 490 | 586 | 586 | 586 | 586 | 586 | 586 | 586 | 586 | 586 | 586 | 586 | 586 | 586 | 586 | 586 | 586 | 586 |
| Rural Location | -0.09 | 0.01 | -0.11 | -0.06 | -0.01 | -0.04 | 0.15 | -0.03 | 0.04 | -0.01 | -0.09 | 0.09 | -0.10 | 1.00 | -0.29 | -0.43 | 0.18 | 0.00 | 0.09 | -0.13 | -0.03 | 0.03 |
|  | 0.06 | 0.81 | 0.01 | 0.22 | 0.76 | 0.31 | 0.00 | 0.41 | 0.30 | 0.72 | 0.03 | 0.03 | 0.02 |  | <.0001 | <.0001 | <.0001 | 0.94 | 0.02 | 0.00 | 0.43 | 0.54 |
|  | 482 | 500 | 512 | 478 | 490 | 586 | 586 | 586 | 586 | 586 | 586 | 586 | 586 | 586 | 586 | 586 | 586 | 586 | 586 | 586 | 586 | 586 |
| Suburban Location | 0.11 | 0.03 | 0.09 | -0.03 | 0.06 | 0.04 | -0.16 | 0.06 | 0.05 | 0.01 | 0.05 | 0.02 | 0.08 | -0.29 | 1.00 | -0.23 | 0.05 | -0.14 | 0.01 | 0.08 | 0.07 | 0.01 |
|  | 0.01 | 0.52 | 0.04 | 0.58 | 0.20 | 0.38 | 0.00 | 0.12 | 0.19 | 0.72 | 0.27 | 0.65 | 0.06 | <.0001 |  | <.0001 | 0.24 | 0.00 | 0.88 | 0.06 | 0.11 | 0.88 |
|  | 482 | 500 | 512 | 478 | 490 | 586 | 586 | 586 | 586 | 586 | 586 | 586 | 586 | 586 | 586 | 586 | 586 | 586 | 586 | 586 | 586 | 586 |
| Urban Location | 0.02 | 0.02 | 0.15 | 0.05 | 0.07 | -0.04 | -0.04 | 0.02 | -0.06 | 0.05 | 0.13 | -0.08 | 0.12 | -0.43 | -0.23 | 1.00 | -0.21 | 0.10 | -0.05 | 0.10 | 0.03 | 0.00 |
|  | 0.64 | 0.60 | 0.00 | 0.29 | 0.11 | 0.38 | 0.40 | 0.61 | 0.12 | 0.24 | 0.00 | 0.07 | 0.00 | <.0001 | <.0001 |  | <.0001 | 0.01 | 0.26 | 0.01 | 0.51 | 0.98 |
|  | 482 | 500 | 512 | 478 | 490 | 586 | 586 | 586 | 586 | 586 | 586 | 586 | 586 | 586 | 586 | 586 | 586 | 586 | 586 | 586 | 586 | 586 |
| White Participants Only | 0.11 | 0.07 | -0.06 | -0.07 | -0.01 | 0.17 | -0.14 | 0.02 | -0.02 | 0.02 | -0.05 | 0.11 | -0.06 | 0.18 | 0.05 | -0.21 | 1.00 | -0.33 | -0.09 | 0.02 | 0.03 | -0.05 |
|  | 0.02 | 0.12 | 0.20 | 0.11 | 0.77 | <.0001 | 0.00 | 0.56 | 0.59 | 0.60 | 0.24 | 0.01 | 0.17 | <.0001 | 0.24 | <.0001 |  | <.0001 | 0.02 | 0.58 | 0.42 | 0.24 |
|  | 482 | 500 | 512 | 478 | 490 | 586 | 586 | 586 | 586 | 586 | 586 | 586 | 586 | 586 | 586 | 586 | 586 | 586 | 586 | 586 | 586 | 586 |
| Non-white Participants Only | -0.16 | -0.03 | -0.03 | 0.04 | -0.08 | -0.23 | 0.32 | 0.10 | -0.04 | -0.17 | 0.02 | -0.10 | 0.01 | 0.00 | -0.14 | 0.10 | -0.33 | 1.00 | 0.10 | -0.08 | -0.02 | 0.10 |
|  | 0.00 | 0.45 | 0.51 | 0.37 | 0.10 | <.0001 | <.0001 | 0.01 | 0.30 | <.0001 | 0.58 | 0.01 | 0.84 | 0.94 | 0.00 | 0.01 | <.0001 |  | 0.01 | 0.06 | 0.66 | 0.02 |
|  | 482 | 500 | 512 | 478 | 490 | 586 | 586 | 586 | 586 | 586 | 586 | 586 | 586 | 586 | 586 | 586 | 586 | 586 | 586 | 586 | 586 | 586 |
| Funding Source: Federal Government/ CDC Funding | 0.04 | 0.06 | 0.08 | 0.13 | -0.03 | -0.11 | 0.13 | 0.02 | 0.03 | -0.02 | -0.03 | -0.01 | 0.07 | 0.09 | 0.01 | -0.05 | -0.09 | 0.10 | 1.00 | 0.03 | 0.03 | -0.12 |
|  | 0.38 | 0.15 | 0.07 | 0.00 | 0.57 | 0.01 | 0.00 | 0.59 | 0.41 | 0.62 | 0.49 | 0.78 | 0.11 | 0.02 | 0.88 | 0.26 | 0.02 | 0.01 |  | 0.51 | 0.52 | 0.00 |
|  | 482 | 500 | 512 | 478 | 490 | 586 | 586 | 586 | 586 | 586 | 586 | 586 | 586 | 586 | 586 | 586 | 586 | 586 | 586 | 586 | 586 | 586 |
| Funding Source: Medicaid and/or Medicare | 0.14 | 0.09 | 0.05 | 0.02 | -0.05 | 0.11 | -0.04 | 0.07 | -0.10 | -0.02 | -0.02 | 0.05 | 0.09 | -0.13 | 0.08 | 0.10 | 0.02 | -0.08 | 0.03 | 1.00 | 0.07 | 0.12 |
|  | 0.00 | 0.05 | 0.24 | 0.67 | 0.26 | 0.01 | 0.36 | 0.11 | 0.02 | 0.63 | 0.58 | 0.23 | 0.03 | 0.00 | 0.06 | 0.01 | 0.58 | 0.06 | 0.51 |  | 0.11 | 0.00 |
|  | 482 | 500 | 512 | 478 | 490 | 586 | 586 | 586 | 586 | 586 | 586 | 586 | 586 | 586 | 586 | 586 | 586 | 586 | 586 | 586 | 586 | 586 |
| Funding Source: State or Local Government Funding and/or State Employee Benefits | 0.04 | 0.12 | 0.12 | 0.01 | 0.00 | -0.13 | -0.03 | 0.03 | 0.26 | 0.06 | -0.09 | 0.02 | 0.04 | -0.03 | 0.07 | 0.03 | 0.03 | -0.02 | 0.03 | 0.07 | 1.00 | -0.04 |
|  | 0.42 | 0.01 | 0.01 | 0.83 | 0.95 | 0.00 | 0.44 | 0.50 | <.0001 | 0.17 | 0.03 | 0.68 | 0.31 | 0.43 | 0.11 | 0.51 | 0.42 | 0.66 | 0.52 | 0.11 |  | 0.33 |
|  | 482 | 500 | 512 | 478 | 490 | 586 | 586 | 586 | 586 | 586 | 586 | 586 | 586 | 586 | 586 | 586 | 586 | 586 | 586 | 586 | 586 | 586 |
| Funding Source: Grant funding | -0.02 | 0.11 | 0.01 | -0.02 | -0.03 | -0.09 | 0.11 | 0.06 | -0.04 | 0.02 | -0.01 | 0.04 | 0.07 | 0.03 | 0.01 | 0.00 | -0.05 | 0.10 | -0.12 | 0.12 | -0.04 | 1.00 |
|  | 0.64 | 0.02 | 0.80 | 0.68 | 0.58 | 0.04 | 0.01 | 0.16 | 0.36 | 0.57 | 0.76 | 0.28 | 0.09 | 0.54 | 0.88 | 0.98 | 0.24 | 0.02 | 0.00 | 0.00 | 0.33 |  |
|  | 482 | 500 | 512 | 478 | 490 | 586 | 586 | 586 | 586 | 586 | 586 | 586 | 586 | 586 | 586 | 586 | 586 | 586 | 586 | 586 | 586 | 586 |
