# Supplementary material for: A structural equation model of CFIR inner and outer setting constructs, organization characteristics, and national DPP enrollment
Source: Implement Sci Commun. 2023 Nov 17;4:142. doi: 10.1186/s43058-023-00522-3 (PMC10657127; doi:10.1186/s43058-023-00522-3)
Supplement: Supplementary file 3 — Additional file 3: Table B. Organization Characteristics and CFIR Item Bi-variates. [file 43058_2023_522_MOESM3_ESM.docx]

**Table B. Organization Characteristics & CFIR Item Bi-variates**

| **Bivariates (Outcome = Enrollment)** | **Parameter Estimate** | **P-value** |
| --- | --- | --- |
| *Continuous Variables* |  |  |
| **Years Delivered** | 0.49 | <.0001 |
| **Lifestyle Coaches at Organization** | 0.21 | <.0001 |
| **Non Lifestyle Coach DPP Staff** | 0.03 | 0.4358 |
| **Number of staff dedicated to National DPP 100%** | 0.52 | <.0001 |
| *Dichotomous Variables* |  |  |
| **DPRP Status** |  |  |
| Full Recognition | 2.12 | <.0001 |
| Pending or Preliminary Recognition | -1.34 | <.0001 |
| No Status/Not Recognized | -1.46 | <.0001 |
| **Organization Size** |  |  |
| Small (0-1,000 people) | -1.05 | .01 |
| Medium (1,000-50,000) | 0.45 | 0.254 |
| Large (Over 50,000) | 1.88 | .001 |
| **Organization Type** |  |  |
| Healthcare/Hospitals | 0.57 | 0.172 |
| Community-based healthcare | 0.08 | 0.847 |
| Community-based organizations | 0.48 | 0.458 |
| Government agencies | -0.23 | 0.673 |
| Academic | -0.88 | 0.266 |
| Health insurers, Employers, Other | 0.56 | 0.197 |
| **Delivery Mode** |  |  |
| In-person (large and small) | 0.47 | 0.226 |
| Virtual (distance, online, hybrid) | 1.21 | 0.005 |
| **Location/Urbanicity** |  |  |
| Rural Location | 0.41 | 0.284 |
| Suburban Location | 1.25 | 0.002 |
| Urban Location | 0.77 | 0.052 |
| **Populations Enrolled** |  |  |
| White-Only | 0.17 | 0.711 |
| Non-White Only | -1.05 | 0.012 |
| **National DPP Funded/Supported By:** |  |  |
| Federal Government/ CDC Funding | 1.93 | <.0001 |
| Medicare or Medicaid | 1.92 | 0.001 |
| State or Local Government Funding | 0.86 | 0.050 |
| State employee coverage benefits | 0.22 | 0.817 |
| State/Local/State Employee | 0.78 | 0.067 |
| Grant funding | 0.93 | 0.019 |
| **CFIR Likert Scale Constructs** |  |  |
| *Inner Setting* |  |  |
| Networks and Communication | 0.79 | 0.001 |
| Culture | 0.75 | 0.004 |
| Implementation Climate | 0.85 | 0.001 |
| Leadership Engagement | 0.49 | 0.037 |
| Available Resources | 0.62 | 0.019 |
| Readiness for Implementation | 0.64 | 0.017 |
| *Outer Setting* |  |  |
| Patient Needs and Resources | 0.75 | 0.005 |
| Cosmopolitanism | 0.80 | 0.001 |
| External Policies and Incentives | 0.76 | 0.002 |
