# Supplementary material for: A structural equation model of CFIR inner and outer setting constructs, organization characteristics, and national DPP enrollment
Source: Implement Sci Commun. 2023 Nov 17;4:142. doi: 10.1186/s43058-023-00522-3 (PMC10657127; doi:10.1186/s43058-023-00522-3)
Supplement: Supplementary file 4 — Additional file 4: Tables C1-3. Structural Equation Model Results. [file 43058_2023_522_MOESM4_ESM.docx]

**Tables C1-3. Structural Equation Model Results**

**Table C1. R-Square**

| **Observed Variable** | **Coefficient (SE)** | **P-value** |
| --- | --- | --- |
| *Latent Variables* | | |
| Inner Setting Implementation | 0.96 (0.03) | <.001 |
| Inner Setting | 0.77 (0.05) | <.001 |
| *Outcome* | | |
| Enrollment | 0.64 (0.05) | <.001 |
| *CFIR Variables* | | |
| Readiness for Implementation | 0.75 (0.03) | <.001 |
| Implementation Climate | 0.76 (0.03) | <.001 |
| Networks and Communication | 0.66 (0.04) | <.001 |
| Culture | 0.71 (0.03) | <.001 |
| Patient Needs & Resources | 0.70 (0.04) | <.001 |
| Cosmopolitanism | 0.50 (0.04) | <.001 |
| External Policies & Incentives | 0.49 (0.04) | <.001 |

**Table C.2 Direct Effects**

| **Outcome** | **Variable** | **Coefficient (SE)** | **P-value** |
| --- | --- | --- | --- |
| *Latent Variables* | | | |
| Inner Setting Implementation | Readiness for Implementation | 0.87 (0.02) | <.001 |
|  | Implementation Climate | 0.87 (0.02) | <.001 |
| Inner Setting | Networks and Communication | 0.81 (0.02) | <.001 |
|  | Culture | 0.84 (0.02) | <.001 |
| Outer Setting | Patient Needs & Resources | 0.84 (0.02) | <.001 |
|  | Cosmopolitanism | 0.71 (0.03) | <.001 |
|  | External Policies & Incentives | 0.70 (0.03) | <.001 |
| CFIR Outcomes | | | |
| Inner Setting | **Outer Setting** | **0.84 (0.03)** | **<.001** |
|  | **Years Delivered** | **-0.09 (0.04)** | **0.04** |
|  | **DPRP Status: Full Recognition** | **0.23 (0.06)** | **<.001** |
|  | **DPRP Status: Pending or Preliminary Recognition** | **0.14 (0.06)** | **0.02** |
|  | DPRP Status: No Status/Not Recognized | 0.01 (0.05) | 0.91 |
|  | Lifestyle Coaches at Organization | 0.07 (0.04) | 0.11 |
|  | Number of Staff Dedicated to National DPP 100% | -0.02 (0.04) | 0.64 |
|  | Organization Size: Small (0-1,000 people) | 0.09 (0.05) | 0.09 |
|  | Organization Size: Medium (1,000-50,000) | 0.00 (0.05) | 1.00 |
|  | Organization Size: Large (Over 50,000) | 0.02 (0.05) | 0.67 |
|  | Organization Type: Community-based healthcare | -0.05 (0.05) | 0.33 |
|  | Organization Type: Community-based organizations | -0.08 (0.04) | 0.06 |
|  | Organization Type: Government agencies | -0.06 (0.05) | 0.21 |
|  | Organization Type: Academic | -0.02 (0.04) | 0.56 |
|  | Organization Type: Health insurers, Employers, Other | -0.04 (0.04) | 0.33 |
|  | Delivery Mode: In-person | -0.02 (0.04) | 0.70 |
|  | Delivery Mode: Virtual | 0.00 (0.04) | 0.94 |
|  | Location: Rural | -0.05 (0.05) | 0.38 |
|  | Location: Suburban | 0.05 (0.05) | 0.31 |
|  | Location: Urban | -0.05 (0.05) | 0.29 |
|  | Population Enrolled White Only | -0.05 (0.04) | 0.27 |
|  | Population Enrolled Non-White Only | 0.06 (0.05) | 0.18 |
|  | Supported By: Federal Government/ CDC Funding | -0.05 (0.04) | 0.26 |
|  | Supported By: Medicare and/or Medicaid | -0.03 (0.04) | 0.53 |
|  | Supported By: State or Local Funding | -0.01 (0.04) | 0.74 |
|  | Supported By: Grant funding | 0.06 (0.04) | 0.15 |
| Inner Setting Implementation | Inner Setting | 0.46 (0.10) | <.001 |
|  | Outer Setting | 0.55 (0.01) | <.001 |
|  | Years Delivered | -0.01 (0.03) | 0.88 |
|  | DPRP Status: Full Recognition | 0.02 (0.05) | 0.69 |
|  | DPRP Status: Pending or Preliminary Recognition | 0.02 (0.05) | 0.66 |
|  | DPRP Status: No Status/Not Recognized | -0.03 (0.04) | 0.39 |
|  | Lifestyle Coaches at Organization | -0.05 (0.03) | 0.13 |
|  | Number of Staff Dedicated to National DPP 100% | 0.00 (0.03) | 1.00 |
|  | Organization Size: Small (0-1,000 people) | -0.02 (0.04) | 0.68 |
|  | Organization Size: Medium (1,000-50,000) | -0.01 (0.04) | 0.84 |
|  | Organization Size: Large (Over 50,000) | -0.04 (0.03) | 0.25 |
|  | Organization Type: Community-based healthcare | 0.06 (0.04) | 0.12 |
|  | Organization Type: Community-based organizations | 0.04 (0.03) | 0.27 |
|  | Organization Type: Government agencies | 0.03 (0.03) | 0.33 |
|  | Organization Type: Academic | 0.01 (0.03) | 0.82 |
|  | **Organization Type: Health insurers, Employers, Other** | **0.08 (0.03)** | **0.01** |
|  | Delivery Mode: In-person | 0.03 (0.03) | 0.26 |
|  | Delivery Mode: Virtual | 0.06 (0.03) | 0.06 |
|  | Location: Rural | -0.04 (0.04) | 0.26 |
|  | Location: Suburban | -0.07 (0.04) | 0.06 |
|  | Location: Urban | -0.07 (0.04) | 0.07 |
|  | Population Enrolled White Only | 0.01 (0.03) | 0.80 |
|  | Population Enrolled Non-White Only | 0.03 (0.03) | 0.30 |
|  | Supported By: Federal Government/ CDC Funding | -0.01 (0.03) | 0.68 |
|  | Supported By: Medicare and/or Medicaid | 0.00 (0.03) | 0.91 |
|  | Supported By: State or Local Funding | 0.02 (0.03) | 0.48 |
|  | Supported By: Grant funding | -0.01 (0.03) | 0.68 |
| Enrollment (scaled /100) | Inner Setting Implementation | 0.29 (0.48) | 0.55 |
|  | Inner Setting | -0.04 (0.25) | 0.86 |
|  | Outer Setting | -0.16 (0.29) | 0.58 |
|  | **Years Delivered** | **0.28 (0.05)** | **<.001** |
|  | DPRP Status: Full Recognition | 0.02 (0.07) | 0.82 |
|  | DPRP Status: Pending or Preliminary Recognition | -0.02 (0.07) | 0.79 |
|  | DPRP Status: No Status/Not Recognized | 0.01 (0.05) | 0.81 |
|  | **Number of Lifestyle Coaches** | **0.47 (0.06)** | **<.001** |
|  | **Number of Staff Dedicated to National DPP 100%** | **0.34 (0.12)** | **0.004** |
|  | Organization Size: Small (0-1,000 people) | 0.03 (0.05) | 0.60 |
|  | Organization Size: Medium (1,000-50,000) | 0.10 (0.05) | 0.05 |
|  | **Organization Size: Large (Over 50,000)** | **0.14 (0.05)** | **0.005** |
|  | Organization Type: Community-based healthcare | -0.07 (0.05) | 0.22 |
|  | Organization Type: Community-based organizations | -0.07 (0.05) | 0.11 |
|  | Organization Type: Government agencies | -0.05 (0.05) | 0.25 |
|  | **Organization Type: Academic** | **-0.13 (0.04)** | **0.001** |
|  | Organization Type: Health insurers, Employers, Other | -0.06 (0.06) | 0.33 |
|  | Delivery Mode: In-person | 0.004 (0.04) | 0.92 |
|  | Delivery Mode: Virtual | 0.03 (0.05) | 0.55 |
|  | **Location: Rural** | **0.21 (0.06)** | **<.001** |
|  | **Location: Suburban** | **0.16 (0.06)** | **0.009** |
|  | **Location: Urban** | **0.16 (0.07)** | **0.015** |
|  | Population Enrolled White Only | -0.03 (0.04) | 0.25 |
|  | **Population Enrolled Non-White Only** | **-0.10 (0.05)** | **0.03** |
|  | Supported By: Federal Government/ CDC Funding | 0.07 (0.04) | 0.10 |
|  | Supported By: Medicare and/or Medicaid | 0.02 (0.04) | 0.58 |
|  | Supported By: State or Local Funding | 0.00 (0.04) | 0.97 |
|  | Supported By: Grant funding | 0.05 (0.04) | 0.25 |

**Table C3. Indirect Effects**

| **Outcome** | **Variable** | **Coefficient (SE)** | **P-value** |
| --- | --- | --- | --- |
| *Indirect Effects* | | | |
| Inner Setting Implementation -> Enrollment  (Indirect 1) | Inner Setting | 0.13 (0.22) | 0.55 |
|  | Outer Setting | 0.16 (.27) | 0.55 |
|  | Years Delivered | -0.001 (0.01) | 0.88 |
|  | DPRP Status: Full Recognition | 0.01 (0.02) | 0.74 |
|  | DPRP Status: Pending or Preliminary Recognition | 0.01 (0.02) | 0.73 |
|  | DPRP Status: No Status/Not Recognized | -0.01 (-0.5) | 0.62 |
|  | Lifestyle Coaches at Organization | -0.01 (0.02) | 0.58 |
|  | Number of Staff Dedicated to National DPP 100% | 0.00 (0.01) | 1.00 |
|  | Organization Size: Small (0-1,000 people) | -0.01 (0.01) | 0.74 |
|  | Organization Size: Medium (1,000-50,000) | -0.002 (0.01) | 0.85 |
|  | Organization Size: Large (Over 50,000) | -0.01 (0.02) | 0.60 |
|  | Organization Type: Community-based healthcare | 0.02 (0.03) | 0.57 |
|  | Organization Type: Community-based organizations | 0.01 (0.02) | 0.60 |
|  | Organization Type: Government agencies | 0.01 (0.02) | 0.61 |
|  | Organization Type: Academic | 0.002 (0.01) | 0.83 |
|  | Organization Type: Health insurers, Employers, Other | 0.02 (0.04) | 0.56 |
|  | Delivery Mode: In-person | 0.01 (0.02) | 0.60 |
|  | Delivery Mode: Virtual | 0.02 (0.03) | 0.57 |
|  | Location: Rural | -0.01 (0.02) | 0.60 |
|  | Location: Suburban | -0.02 (0.03) | 0.57 |
|  | Location: Urban | -0.02 (0.04) | 0.57 |
|  | Population Enrolled White Only | 0.002 (0.01) | 0.82 |
|  | Population Enrolled Non-White Only | 0.01 (0.02) | 0.61 |
|  | Supported By: Federal Government/ CDC Funding | -0.004 (0.01) | 0.73 |
|  | Supported By: Medicare and/or Medicaid | 0.001 (0.01) | 0.91 |
|  | Supported By: State or Local Funding | 0.01 (0.01) | 0.65 |
|  | Supported By: Grant funding | -0.004 (0.01) | 0.73 |
| Inner Setting -> Enrollment  (Indirect 2) | Outer Setting | -0.04 (0.21) | 0.86 |
|  | Years Delivered | 0.004 (0.02) | 0.86 |
|  | DPRP Status: Full Recognition | -0.01 (0.06) | 0.86 |
|  | DPRP Status: Pending or Preliminary Recognition | -0.01 (0.04) | 0.86 |
|  | DPRP Status: No Status/Not Recognized | 0.00 (0.003) | 0.92 |
|  | Lifestyle Coaches at Organization | -0.003 (0.02) | 0.86 |
|  | Number of Staff Dedicated to National DPP 100% | 0.001 (0.01) | 0.87 |
|  | Organization Size: Small (0-1,000 people) | -0.004 (0.02) | 0.86 |
|  | Organization Size: Medium (1,000-50,000) | 0.00 (0.002) | 1.00 |
|  | Organization Size: Large (Over 50,000) | -0.001 (0.01) | 0.87 |
|  | Organization Type: Community-based healthcare | 0.002 (0.17) | 0.86 |
|  | Organization Type: Community-based organizations | 0.004 (0.02) | 0.86 |
|  | Organization Type: Government agencies | 0.003 (0.02) | 0.86 |
|  | Organization Type: Academic | 0.001 (0.01) | 0.87 |
|  | Organization Type: Health insurers, Employers, Other | 0.002 (0.01) | 0.61 |
|  | Delivery Mode: In-person | 0.001 (0.004) | 0.87 |
|  | Delivery Mode: Virtual | 0.00 (0.002) | 0.94 |
|  | Location: Rural | 0.002 (0.01) | 0.87 |
|  | Location: Suburban | -0.002 (0.01) | 0.86 |
|  | Location: Urban | 0.002 (0.01) | 0.86 |
|  | Population Enrolled White Only | 0.002 (0.01) | 0.86 |
|  | Population Enrolled Non-White Only | -0.003 (0.02) | 0.86 |
|  | Supported By: Federal Government/ CDC Funding | 0.002 (0.01) | 0.86 |
|  | Supported By: Medicare and/or Medicaid | 0.001 (0.01) | 0.87 |
|  | Supported By: State or Local Funding | 0.001 (0.004) | 0.88 |
|  | Supported By: Grant funding | -0.003 (0.02) | 0.86 |
| Inner Setting -> Inner Setting Implementation -> Enrollment  (Indirect 3) | Outer Setting | 0.11 (0.19) | 0.55 |
|  | Years Delivered | -0.01 (0.02) | 0.57 |
|  | DPRP Status: Full Recognition | 0.03 (0.05) | 0.56 |
|  | DPRP Status: Pending or Preliminary Recognition | 0.02 (0.03) | 0.56 |
|  | DPRP Status: No Status/Not Recognized | 0.001 (0.05) | 0.81 |
|  | Lifestyle Coaches at Organization | 0.01 (0.02) | 0.58 |
|  | Number of Staff Dedicated to National DPP 100% | -0.002 (0.01) | 0.71 |
|  | Organization Size: Small (0-1,000 people) | 0.01 (0.05) | 0.60 |
|  | Organization Size: Medium (1,000-50,000) | 0.00 (0.01) | 1.00 |
|  | Organization Size: Large (Over 50,000) | 0.003 (0.01) | 0.73 |
|  | Organization Type: Community-based healthcare | -0.01 (0.01) | 0.61 |
|  | Organization Type: Community-based organizations | -0.01 (0.02) | 0.57 |
|  | Organization Type: Government agencies | -0.01 (0.01) | 0.59 |
|  | Organization Type: Academic | -0.003 (0.01) | 0.68 |
|  | Organization Type: Health insurers, Employers, Other | -0.01 (0.01) | 0.61 |
|  | Delivery Mode: In-person | -0.002 (0.01) | 0.74 |
|  | Delivery Mode: Virtual | 0.00 (0.01) | 0.94 |
|  | Location: Rural | -0.01 (0.01) | 0.62 |
|  | Location: Suburban | 0.01 (0.01) | 0.61 |
|  | Location: Urban | -0.01 (0.01) | 0.60 |
|  | Population Enrolled White Only | -0.01 (0.01) | 0.60 |
|  | Population Enrolled Non-White Only | 0.01 (0.02) | 0.58 |
|  | Supported By: Federal Government/ CDC Funding | -0.01 (0.01) | 0.60 |
|  | Supported By: Medicare and/or Medicaid | -0.003 (0.01) | 0.66 |
|  | Supported By: State or Local Funding | -0.002 (0.01) | 0.77 |
|  | Supported By: Grant funding | 0.01 (0.02) | 0.58 |
